# Latest Advances in the Management of Pediatric Gastrointestinal Stromal Tumors

**DOI:** 10.3390/cancers14204989

**Published:** 2022-10-12

**Authors:** Marta Andrzejewska, Jakub Czarny, Katarzyna Derwich

**Affiliations:** 1 Faculty of Medicine, Poznan University of Medical Sciences, 61-701 Poznan, Poland or; 2 Department of Pediatric Oncology, Hematology and Transplantology, Institute of Pediatrics, Poznan University of Medical Sciences, 60-355 Poznan, Poland

**Keywords:** gastrointestinal stromal tumor, pediatric GIST, kinase inhibitors, molecular diagnostics

## Abstract

**Simple Summary:**

Though gastrointestinal stromal tumor is the most common mesenchymal neoplasm of the gastrointestinal tract, it is a rare entity among pediatric patients. It is usually characterized by a different molecular biology, histology and clinical course. Therefore, different handling of pediatric GIST is needed. Herein, we review the latest updates to the management of pediatric gastrointestinal tumors with a particular focus on the advances in molecular biology of the disease and emerging treatment with kinase inhibitors that could serve as targeted therapy.

**Abstract:**

Gastrointestinal stromal tumor is the most common mesenchymal neoplasm of the gastrointestinal tract, usually found in elderly adults. It is infrequent among pediatric patients and usually differs biologically from adult-type diseases presenting mutations of *KIT* and *PDGFR* genes. In this population, more frequent is the wild-type GIST possessing *SDH*, *TRK*, *RAS*, *NF1* mutations, among others. Both tumor types require individualized treatment with kinase inhibitors that are still being tested in the pediatric population due to the different neoplasm biology. We review the latest updates to the management of pediatric gastrointestinal tumors with a particular focus on the advances in molecular biology of the disease that enables the definition of possible resistance. Emerging treatment with kinase inhibitors that could serve as targeted therapy is discussed, especially with multikinase inhibitors of higher generation, the effectiveness of which has already been confirmed in the adult population.

## 1. Introduction

Gastrointestinal stromal tumor (GIST) arises from Cajal cells present in the gastrointestinal tract. It is the most common mesenchymal tumor arising in this localization; however, it is an extremely rare entity in the pediatric population. Only up to 2% of GISTs are diagnosed in children and adolescents [1]. Usually, pediatric GIST are present as sporadic tumors and do not harbor c-KIT or PDGFRA (platelet-derived growth factor receptor α) mutations [2]. They may be associated with abnormalities of the *SDH* gene encoding gene (including *SDHB*) or the *IGF-1R* gene encoding insulin-like growth factor 1 receptor [3]. Histopathologically, the tumor tends to have epithelioid or mixed histology, which is different from classical spindle cell adult histology [3,4]. The tumor is often localized in the stomach, yet it may be present in any gastrointestinal localization or outside it: cases of the peritoneal, abdominal wall or pulmonary GISTs are reported in the literature, among others. Clinically, it usually manifests with anemia, bleedings or occult stool blood and its course may be indolent, yet it also metastasizes more often than in adults [3]. However, the rate has not been compared objectively and adequately, and this remark is based on case series with a long follow-up of the patients.

The basic management of pediatric GIST involves a slightly modified imaging diagnostic approach as the use of computed tomography is discouraged, favoring the use of MRI, USG and PET, therefore lowering the child’s exposure to ionizing radiation, as well as ensuring adequate diagnostics [5,6]. An incisional biopsy should be undertaken to validate the primary diagnosis. Then, R0 surgical procedure without lymph node removal is performed [7] with curate intention. It is vital to perform genetic testing on the specimen to get to know its biology and possible mutations to personalize further treatment if needed. It also allows for the classification of GIST: some children have an adult-type tumor that possesses KIT or PDGFRA mutations, which may allow for the use of imatinib (especially exon 9 or 11 KIT mutations that are sensitive to imatinib treatment) [8]. Pediatric-type GIST may be treated with different agents compatible with the exact genetic alterations [9,10].

## 2. Molecular Diagnostics of Pediatric GIST and Its Selected Consequences

There is a great need to state the molecular type of pediatric GIST, detecting genetic abnormalities, which is possible with DNA and RNA sequencing and immunohistochemistry methods [11,12,13,14]. As the single-molecule molecular inversion probe (smMIP) technology allows easy adaptation of panels, detection of the clue mutations in GIST can proceed with smMIP-based next-generation sequencing (NGS) panels [15]. However, whole exome sequencing (WES) and transcriptome analysis on formalin-fixed paraffin-embedded tissue allows the more complex analysis of the biology of GIST [16] and to apply targeted therapy when it comes both to the substance and its dose. The whole laboratory process should be strongly combined with the appropriate genetic counseling of patients and their families before embarking on a surveillance program.

### 2.1. NTRK Genetics in Wild-Type Pediatric GIST

Around 85% of pediatric GISTs are wild-type (WT-GIST), which makes up for 10% of all diagnosed tumors of this type. They are often characterized by mutations or silencing of four genes encoding the subunits of the SDH enzyme complex [7]. Moreover, the analyses show the frequent presence of *NTRK*/*TRK* (neurotrophic tyrosine kinase receptor) mutations (*NRTK1, NTRK2, NTRK3*), including fusions (e.g., *ETV6::NTRK3*, *TPM3::NTRK1*, *TPR::NTRK1*, *LMNA::NTRK1*, *SPECC1L::NTRK3*), in cases of WT-GISTs (5–25%) [17,18]. They can lead to different manifestations but also determine a specific treatment method. In parallel, they are characterized by the lack of canonical KIT, PDGFRA, SDHx or RAS pathway components (*KRAS*, *BRAF*, *NF1*) alterations, which eliminates the possibility of the use of a range of multikinase inhibitors [13]. Therefore, it is necessary to individualize treatment and apply other types of kinase inhibitors.

Thanks to the good antitumor efficacy of NTRK inhibitors, both in adults and children, there is a great need for individualized prognosis of pediatric GIST with genetic studies, including NTRK genes fusion status study, e.g., using hybrid capture DNA-based targeted panels (UCSF500 Cancer Gene Panel and OncoPanel) that include probes for exons and select introns of *NTRK1, NTRK2*, and *NTRK3*, as well as *ETV6* exonic and intronic probe [17,19,20]. The World Sarcoma Network recommends *NRTK* testing in locally advanced and unresectable or metastatic sarcoma, with very high priority, classifying WT-GIST as sarcoma with low frequency of NTRK fusions. In the case of positive Pan-TRK immunochemistry, massive parallel sequencing is recommended to consider TRK inhibitors. Otherwise, additional *NTRK* testing is unnecessary. However, immunohistochemistry is not advised when myogenic and neural differentiation is present in histological diagnosis due to the high rate of false positivity [21].

### 2.2. FGFR Genetics in Wild-Type Pediatric GISTs

Some GISTs are connected with *FGFR* (fibroblast growth factor receptor) genetic abnormalities, including oncogenic fusion *FGFR1::HOOK3*, *FGFR1::TACC1*, activating *FGFR1* missense mutations p.K656E and p.N546K, FGF4 overexpression; FGF2 overexpression or gain in FGFR2 in imatinib-resistant GIST cells [13,22,23,24,25]. Therefore, overcoming imatinib resistance with FGF pathways inhibitors, e.g., via the molecular mechanism of sensitization to DNA damaging agents, for example, DNA-topoisomerase II inhibitors can be a novel pathway in studies. It is possible thanks to the attenuation of DNA double-strand break repair after the application of a selective FGFR inhibitor. Therefore, doxorubicin treatment with the standard chemotherapeutic agents (i.e., doxorubicin) increases apoptosis [26,27].

### 2.3. RAS/NF1-Related Genetics in Wild-Type Pediatric GISTs

Searching for mutations in GIST remains necessary not only to predict a tumor’s malignancy but also to find molecular treatment targets, e.g., BRAF mutations to use BRAF inhibitors (e.g., dabrafenib) or *NF1* mutations to use MEK inhibitors.

It is worth remembering that due to the NF1 (neurofibromin 1) participation in RAS-mediated pathways and possible RAS-related mutations in GISTs, *NF1*-mutant GIST SDHB IHC retained (+) is one of the WT-GIST. It is mainly located in a small intestine, however small, and multicentric, with low mitotic activity and a good prognosis. Therefore, it is worth monitoring children with RASopathies to detect the possible occurrence of GIST soonest because of the poor prognosis of metastatic disease and to treat them more efficiently with RAS-related inhibitors due to the potential resistance to imatinib and sunitinib. Likewise, BRAF-mutant and RAS-mutant SDBH IHC retained (+) GIST can also be detected independently of age (like *NF1*-mutants) but characterized more often with spindle cell morphology. The clinical course of this type of WT-GIST varies among the patients [28]. *BRAF* mutations in GISTs don’t present prognostic value themselves but remain a possible target for therapeutic options to control disease, especially in the case of an advanced stage [29].

### 2.4. SDH Genetics in Wild-Type Pediatric GISTs

Considering the familial GIST type (hereditary GIST) associated with *SDH* mutations, the interview and active screening remain beneficial to be able to suspect Carney–Stratakis syndrome (CSS). Mutations in the mitochondrial tumor suppressor gene pathway involve the succinate dehydrogenase subunits SDHA (most frequently), SDHD, SDHC (c.24delC in the exon 1 of *SDHC* and resulting in the frameshift variant p.His8GlnfsTer39, with a mutant allele burden >90%) and SDHB (c.287-1G > C in intron 3 with a mutant allele burden >95%) [30,31].

However, more characteristic of pediatric GISTs is the type with SDHB IHC loss, usually located in the stomach. Their traits are multinodular/plexiform growth, joint lymphatic invasion and lymph node metastases, and unpredictable behavior, including indolent clinical course (also in a metastatic state) [30,31,32].

SDH-deficient GISTs can be associated with Carney’s triad syndrome, a non-heritable syndrome related to GIST, pulmonary chondroma, and paraganglioma, mainly seen in girls and young women. The potential time from GIST diagnosis to the appearance of other components visible on CT may be more than 8 years, but possibly even 3 decades. Therefore, pediatric patients should receive regular clinical examinations to detect possible extra-adrenal paraganglioma and pulmonary chondroma rapidly. During the molecular diagnostic process of pediatric GIST, associations with hereditary paraganglioma-pheochromocytoma syndromes are worth searching [33,34].

It has been shown in some tumors that SDH complex is inactivated by hypoxia-inducible factor (*HIF*), which is understandable given its role in aerobic respiration. *HIF* is not degraded and is stabilized due to earlier succinate accumulation. Inactivated SDH thus promotes increased angiogenesis via the pseudo-hypoxic patchway. *VEGF* (vascular endothelial growth factor), *GLUT1* (glucose transporter 1) and *M-CSF* (macrophage colony-stimulating factor) are also described to be upregulated. These all features serve as potential targets for the subsequent targeted therapy [35,36].

### 2.5. New Biomarkers of High-Risk GIST

HAND1 presents higher expression in metastatic or high-risk KIT-mutant GIST and supports KIT expression. Moreover, HAND1(+) tumor cells express proliferative markers. HAND1, expressed solely in small intestine interstitial cells of Cajal (ICCs), is a marker of aggressive behavior of GIST in the gastrointestinal tract out of the small intestine in contrast to S100A associated strictly with small intestine origin. BARX1 expression is restricted to gastric ICCs and is positive in the PDGFRA-mutated and WT-GIST groups, which also show more indolent clinical presentation and, usually, no recurrence. Thus, HAND1 and BARX1 expression in tumor cells might determine prognosis and the need for adjuvant imatinib therapy because of shorter progression-free survival in such GIST [37].

## 3. Perspectives in Pharmacological Management of Pediatric GIST with Use of Multikinase Inhibitors

### 3.1. Imatinib

Imatinib is still the most widely used agent in first-line therapy for either KIT/PDGFR or wild-type pediatric GIST. Imatinib is often administered after R1 surgery as adjuvant treatment (in the high-risk group) and before surgical treatment as neoadjuvant treatment with an additional adjuvant dose in the high-risk group. It is also useful in the management and prolonging the overall survival of patients with metastatic disease. Despite all that, researchers seek other favorable options for pediatric patients. The rarity of this type of tumor makes it difficult to construct a proper clinical trial, which is additionally ethically concerning in pediatric patients [2,38,39,40,41]. However, one of the NCT01738139 trial’s goals is to check the best dose of ipilimumab [42,43,44] and imatinib mesylate in treating patients with GIST over 15 years old.

### 3.2. Sunitinib

In the case of imatinib-resistant tumors, the second choice is usually sunitinib, a multikinase inhibitor of higher potency, especially in KIT(wt) GIST. The starting and maximum tolerated dose in children is 15 mg/m^2^ (study ADVL0612; NCT00387920; A6181196; NCT01396148), yet pharmacodynamic simulations show 25 mg/m^2^ dose as more efficient, providing the drug’s plasma concentrations similar to those observed in the adult population with 50 mg/m^2^ dose. The ongoing trial in phase I/II (NCT01396148) shows the tolerable dose of sunitinib in first-line therapy to be a minimum of 20 mg/m^2^ every 4 weeks with a rest period of 2 weeks. This provides an acceptable safety profile as observed in adults on 50 mg/m^2^ dose and the same schedule, and 5.8 months median PFS (progression-free survival). According to European and US records, only 9 children were treated with sunitinib until 2017 [1]. They were administered sunitinib 50 mg/day on a 4/2 schedule. An alternative was a continuous treatment with 37.5 mg/day. In the NCT01396148 study, stabilization of the disease was observed in three patients (50%) by day 15, cycle 1, with no additional accumulation across cycles [45,46,47,48,49,50]. Moreover, Forsythe et al. postulated MCV elevations during treatment with sunitinib may be an apparent drug-related epiphenomenon and possible valuable marker for therapeutic drug monitoring and treatment adherence indicator for pediatric and adult patients with no adverse events, but they considered it as an indicator of therapeutic toxicity [51].

### 3.3. Regorafenib

Another accessible alternative for imatinib-resistant tumors is the administration of regorafenib, a multikinase inhibitor targeting VEGFR (vascular endothelial growth factor), TIE-2 (angiopoietin-1 receptor), FGFR, PDGFR, KIT, RET oncogene and RAF kinases among others. While it is well-known in adults, it was only administered in a limited number of pediatric GIST cases. Its use with a dose of 160 mg/day 3 weeks in a month is beneficial. Moreover, regorafenib has been observed to possess radio- and chemosensitizing outcomes, especially in PDGFR-mutated GIST. It can also be beneficial in *KIT*-mutated tumors. In preclinical GIST models carrying a mutated *KIT* oncogene, partial regressions were observed after regorafenib application. Moreover, in vivo regorafenib exhibits significant antitumor activity in various pediatric malignancies, independent of histological tumor type, through inhibition of angiogenesis [52,53,54,55,56,57].

### 3.4. Avapritinib

An emerging drug dedicated to patients with a mutation in exon 18 of the PDGFRA gene (such as D842V) is avapritinib, applied in the NCT03862885 trial in patients with locally advanced unresectable or metastatic GIST over 16 years old. One of the other main inclusion criteria is the foregoing reception of 3 or more TKI therapies, including imatinib. Some higher-generation tyrosine kinase inhibitors are less effective in adult GISTs, e.g., nilotinib [58], though it hasn’t been proven in pediatric GISTs.

### 3.5. Vandetanib and Guadecitabine

Vandetanib, a small molecule inhibitor of VEGFR2, EGFR (epidermal growth factor), and RET is one of the new potential drugs in children and adults with succinate dehydrogenase deficient (dSDH) GIST. However, neither partial nor complete responses nor meaningful changes in the tumor growth or density rate were observed (median number of cycles 4, range 2–18, median PFS of participants-5,1 months). Regardless, improved molecular characterization of dSDH GISTs (including mutations, promoter methylation status or global DNA methylation status (e.g., hypermethylation), will be important in designing future therapeutic trials to apply such type of inhibitor [59]. In this type of pediatric GISTs, loss of activity of SDH in the Krebs cycle and, therefore, accumulation of succinate inhibiting α-ketoglutarate-dependent dioxygenases leads to DNA hypermethylation. Therefore, one of the new potential chemotherapeutics, guadecitabine, which is a small molecule DNA methyltransferase inhibitor, can reverse DNA hypermethylation in tumors with Krebs cycle abnormalities. Phase II study (NCT03165721) showed good tolerance by the majority of patients with SDH-deficient GIST, however, no complete or partial responses were observed [32].

### 3.6. NRTK Inhibitors

When it comes to TRK-fusion GISTs, first studies prove the good antitumor efficacy, a high and durable clinical response rate and good toleration of treatment with larotrectinib and entrectinib, NTRK inhibitors, both in adults and children, including recurrent or refractory neoplasms (e.g., NCT02122913, NCT02637687 and NCT02576431 involved 3 cases of GIST) [60,61].

### 3.7. Sorafenib and Other Multikinase Inhibitors

Several targeted therapies also play the role of multikinase inhibitors [37], such as sorafenib [40,62,63,64], avapritinib [65,66,67], ponatinib [68], whose effectiveness is confirmed in adult GIST treatment; however, there has been any cohort evidence in the literature of clinical effectiveness in the pediatric population. Sorafenib has been used with excellent efficacy in 2 patients with BRAF-mutated wildtype GIST resistant to imatinib, sunitinib and regorafenib and has proved ineffective in BRAF V600E mutated GIST [69]. Based on a case report published by Brinch et at., it may be effective in patients with D842 PDGFRA exon 18 mutations that are usually insensitive to imatinib [64]. Relying on the experience of its use in other pediatric tumors, it may be used in pediatric GIST.

### 3.8. Ripretinib

Finally, the effect of the recent development of KIT/PDGFRA inhibitors is ripretinib (formerly DCC-2618), whose effectiveness in inhibition of a wide range of KIT mutants in patients with drug-resistant GISTs has been confirmed in preclinical cancer models and preliminary clinical data [70]. PFS as a primary outcome measure is currently verified in a phase 3 study (NCT03353753 trial) in advanced adult GISTs treated with prior anticancer therapies. Moreover, multicenter, global, randomized, open-label Phase III INTRIGUE (NCT03673501 trial) compares the efficacy of ripretinib in comparison with sunitinib in advanced adult GISTs after treatment with imatinib.

### 3.9. Treatment of SDH-Mutated and Carney-Stratakis Syndrome Associated GIST

Succinate dehydrogenase (*SDH*) deficiency is one of the possible genetic alterations in wild-type pediatric GIST that is associated with this entity the most frequently. Such type of tumor should discard the use of imatinib. However, the patients could benefit from using the aforementioned kinase inhibitors with anti-angiogenic activity, i.e., sunitinib, vandetanib, sorafenib or regorafenib. The existing evidence is limited and based only on individual cases of patients with *SDH*-deficient tumors enrolled in more extensive trials. Unfortunately, they indicate limited efficacy of sunitinib, regorafenib, nilotinib and a lack of efficacy of vandetanib in this type of tumor [59,71,72,73,74]. Ganjoo et al. report a patient that took pazopanib showed long-lasting disease control [75]. Morever, such patients rarely profit from the neoadjuvant therapy [76]. There have been two clinical trials adjusted for SDH-deficient GIST among other neoplasms, NCT02071862 involving glutaminase inhibitor CB-839 and NCT03165721 with guadecitabine (SGI-110), though both were cessated and no results have been published.

Due to the rarity of this neoplasm in children, the scarcity of available data and the ethical considerations of clinical trials in the pediatric population, the aforementioned reviewed data relies on a tumor-agnostic approach. Unfortunately, there has not been a large multicenter clinical trial or head-to-head trial that could assess the actual effectiveness of these agents in children.

**Figure 1:**
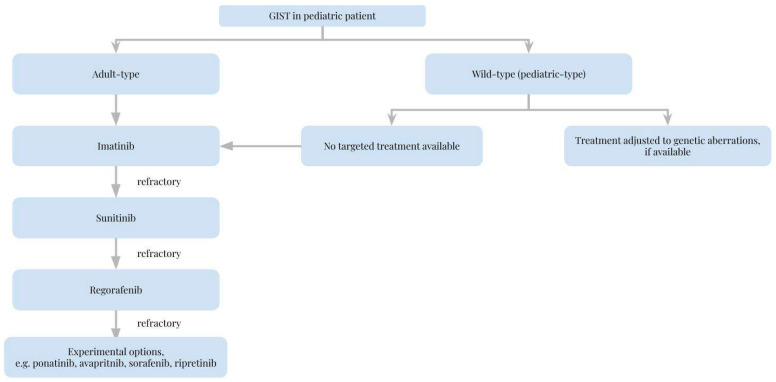
Choice of kinase inhibitors in the management of pediatric gastrointestinal stromal tumor.

Table 1 lists the agents that are or might be used in pediatric GISTS and their molecular targets and provides short comments on their use. Figure 1 provides a suggested algorithm for the process of choosing a kinase inhibitor for the treatment of pediatric GIST.

## 4. Updates to Surgical Approach

### 4.1. Current Surgical Standards

Overall, the last years did not bring any major advances in the surgical approach to pediatric GISTs. Surgical resection remains the main treatment method that should be undertaken after the suspicion of GIST. However, it can be performed as wide local excision to total gastrectomy. Tumor size >5 cm, high mitotic index, and spindle morphology predict mortality. Pediatric GISTs have a more favorable prognosis in comparison to adult GISTs [4,78,79,80]. Frequent recurrence of GISTs is present despite complete resection and multiple surgeries.

Approximately 30% of the pediatric GISTs develop regional lymph node metastases, which differs from GISTs in adults (≤2% of GIST with lymph node metastases). Therefore, some parts of pediatric GIST of high-risk lymph node metastases may require dissection of lymph nodes. A precise definition of this population should be discussed as this is not a common standard now [31,81,82]. Other gastrointestinal tumors can be mistaken in some cases as GIST [82] or found accidentally, e.g., in the material from an appendectomy [83].

No guidelines concerning the surgical approach to differently mutated GIST have been developed to date [74]. The recommended option of pediatric GISTs surgical treatment is a limited operation with wedge resection, as obtaining negative gross margins is indicated. Though, the laparoscopic surgery of gastric GIST and pediatric WT-GIST remains possible [5,84,85]. Simple enucleations generate a high risk of recurrence. Matsumoto et al. proposed a laparoscopic-endoscopic cooperative surgery-related procedure for tumor resection transorally using the non-exposed endoscopic wall-inversion surgery in the following steps: marking around the tumor on the mucosal and serosal surfaces, performing a submucosal injection of sodium hyaluronate with indigo carmine dye and afterward, circumferential seromuscular dissection with suture closure under the laparoscopic vision and finally a circumferential muco-submucosal incision under gastric endoscopic vision. Tumor resection can proceed from every position in the stomach. However, closure of the esophagogastric junction or the pyloric ring may be difficult [86]. The amount of tissue required to make the GIST diagnosis is 1 cm^3^ (about 5–10 core-needle biopsy specimens) [5]. EUS (endoscopic ultrasound)-guided fine-needle with 95% ethanol injection (FNI) therapy of GISTs in adults is an effective treatment method [87,88], however, its application in pediatric GISTs has not been set down yet.

### 4.2. Biopsy of GIST

One of the possible pediatric GIST symptoms, especially in the stomach, is bleeding from a tumor mass because of the central vessel bulging into the lumen. It also makes up the negative predictive factor for pediatric GIST treatment as so-called tumor rupture that can be defined by tumor fracture or spillage, blood-stained ascites, microscopic infiltration of an adjacent organ, intralesional dissection or piecemeal resection or incisional biopsy [89,90,91,92,93]. Jakob et al. performed a systematic review to determine the risk of needle tract seeding and abdominal recurrence of GIST after a pretreatment biopsy. Even though GIST is considered to be a fragile, easily-rupturing tumor, it has been found that EUS-FNA-guided biopsy does not augment the risk of the aforementioned events [94].

### 4.3. Benefits from Extensive GIST Resection

However, due to the indolence of pediatric WT-GISTs and their biology, there is no improvement in event-free survival, even after extensive or serial resections and in the presence of disease progression or recurrence. Therefore, resections of WT-GISTs should be limited to the initial procedure unless symptoms, like obstruction or bleeding, are present [84].

### 4.4. Hyperthermic Intraperitoneal Chemotherapy in Pediatric GIST

As peritoneal metastases may be present in pediatric GISTs, one of the methods used to remove the visible macroscopic disease is HIPEC (hyperthermic intraperitoneal chemotherapy) with complete cytoreduction (CRS) that can be proceeded even in infant treatment [95]. There is still little data concerning the use of HIPEC not only in pediatrics, but also adult patients, especially in respect to GIST, though its use in peritoneal sarcomatosis is gaining more attention. It was also used in palliative GIST-related ascites treatment. Importantly, HIPEC procedure in pediatric patients was linked with a smaller mortality and mortality as in adult population [96,97,98]. However, owing to the safety of the procedure patient selection and technique modification is necessary, and all important considerations were summarized by Garnier et al. CRS preceding HIPEC makes up the key factor in survival [95]. It is unknown, however, which cytostatic agent should be used in HIPEC treatment of GIST—there are reports on the use of mitomycin C.

### 4.5. Possible Future Amendments

Future development in the surgical management of GIST may be characterized by the search for the application of fluorescence markers to improve surgical procedures. To date, indocyanine green has proved to be inefficient in this matter [99]. Another promise is the future widespread use of 3D planning and augmented reality to better plan and execute the surgery, especially in tumors of difficult localization, including vessel- and neuroinvasive tumor. Until now, there has been no report of such practice in pediatric gastrointestinal stromal tumor, though this has already been used in different types of sarcomas [100,101,102].

## 5. Conclusions

**Figure 2:**
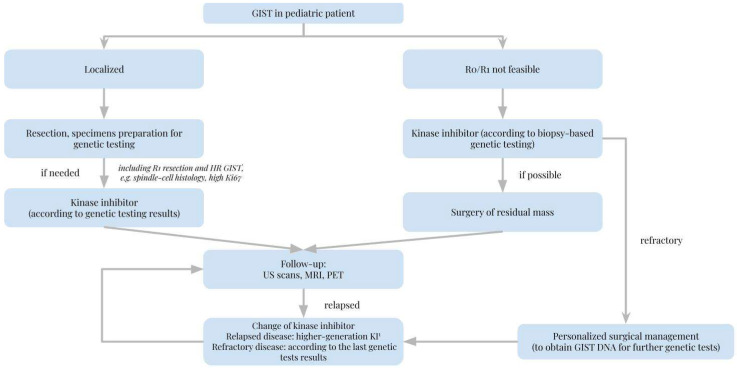
Management algorithm when a gastrointestinal stromal tumor in a pediatric patient is diagnosed.

Pediatric GISTs are characterized by distinct biology that is still under numerous studies due to scarce study material. Due to little evidence from multicenter studies, we propose an oncoagnostic approach that could be of great importance. This entity requires complex genetic testing combined with histopathological diagnosis to apply the treatment properly. To the best of our knowledge, despite few studies, such a complex approach and tailoring the treatment to the molecular goal may lead to remission. We depict the importance of genetic studies that should contain not only *KIT* or *PDGFR* genes but also *TRK*, *FGFR*, *RAS*, *SDH* genes and potentially genes encoding for other multikinase inhibitors targets. The establishment of the NGS panel or even WES dedicated to pediatric GIST would enable the possibility of stating the prognosis and, if needed, administration of proper multikinase inhibitor (of proper generation). While imatinib remains the first-line agent for pediatric GIST, it should be recognized it is not suitable for every patient. Medications of higher generation (such as sunitinib, regorafenib) are important in overcoming imatinib resistance, though, in the future other targeted therapies (such as sorafenib, ponatinib, avapritinib) may play a significant role in the treatment of adult-type GISTs in children. As most pediatric GISTs are wild-type, non-standard kinase inhibitors, e.g., larotrectinib, entrectinib, could be applied in such cases. Nevertheless, there is still limited data regarding their administration, especially in pediatric patients. Finally, when it comes to surgical treatment in pediatric GISTs, we propose the application of laparoscopic surgery with lymph nodes excision as the method of choice with the MRI, USG and PET imaging control. Although pediatric GISTs belong to rare children’s neoplasms, unified diagnostics and treatment guidelines are necessary to systematize the approach in gastrointestinal stromal tumors in pediatric patients. We encourage a tumor agnostic approach, especially in wild-type/pediatric-type GIST, proposing a provisional management algorithm (Figure 2).

## Figures and Tables

**Table 1:** Current application and perspectives in targeted therapies in pediatric GISTs treatment.

Drug Substance	Molecular Target	Comments	Reference
Imatinib	PDGF, SCF, KIT, C-KIT, ABL (including BCR-ABL fusion)	First-line treatment after R1 surgery (including high-risk group); if needed- neoadjuvant treatment	[2,38,39,40,41]
Sunitinib	PDGFR-α, PDGFR-β, VEGFR1, VEGFR2, VEGFR3, KIT, FLT3, CSF-1R, RET	Usually the second-line treatment of imatinib-resistant GIST; leads to the tumor stabilization	[1,45,46,47,48,49,50]
Regorafenib	VEGF-1, VEGF-2, VEGF-3, VEGFR-1, VEGFR-2, VEGFR-3, TIE-2, KIT, RET, RAF-1, B-RAF, PDGFR(-β), FGFR-1, EGFR	Beneficial for KIT-mutated and PDGFR-mutated GIST, including radio- and chemosensitization in vitro; anti-angiogenic effect (limited data)	[52,53,54,55,56,57]
Nilotinib	BCR-ABL, KIT, LCK, EPHA3, EPHA8, DDR1, DDR2, PDGFRB, MAPK11, ZAK	Less effective in adult GIST than imatinib without evidence in the pediatric population	[58]
Vandetanib	VEGFR-2, VEGFR-3 (weak interaction), VEGF, EGFR(-3), EGF, RET	No meaningful changes observed after application so far	[59]
Larotrectinib	TRK A, B, C	High and durable clinical response rate, good toleration of treatment	[60,61]
Entrectinib	TRK A, B, C, ROS1, ALK	High and durable clinical response rate, well-tolerated	[60,61]
Sorafenib	RAF kinases (C-RAF > B-RAF), PDGFR kinases, PDGFRβ, VEGFR-1, VEGFR-2, VEGFR-3, FLT-3, C-KIT, RET, induction of autophagy	Effective in adult GIST treatment but ineffective in V600E *BRAF* mutation, no evidence of efficacy in pediatric GIST	[40,62,63,64,69]
Avapritinib	KIT, PDGFRA mutant (such as D816 V KIT and D842 V PDGFRA)	Effective in adult GIST treatment, no evidence of efficacy in pediatric GIST treatment, ongoing NCT03862885 trial with early access for avapritinib in patients >16 years old with locally advanced unresectable or metastatic GIST	[65,66,67]
Ponatinib	ABL (BCR-ABL mutants), VEGFR, PDGFR, EGFR, SRC kinase, KIT, RET, FLT3	Effective in adult GIST treatment, no evidence of efficacy in pediatric GIST treatment yet	[68,77]
Ipilimumab	CTLA-4	Good safety, limited data about the treatment efficacy in combination with other multikinase inhibitors, ongoing NCT01738139 trial in patients >15 years old	[42,43,44]
Ripretinib	KIT and PDGFRA kinases	Effective inhibition of a wide range of KIT mutants in patients with drug-resistant GISTs (preclinical models, phase III trial finished)	[70]

## Data Availability

Not applicable.
